# Supplementary material for: HM-DyadCap – capture and mapping of 5-hydroxymethylcytosine/5-methylcytosine CpG dyads in mammalian DNA
Source: Nucleic Acids Res. 2026 Apr 23;54(8):gkag389. doi: 10.1093/nar/gkag389 (PMC13103736; doi:10.1093/nar/gkag389)
Supplement: gkag389_Supplemental_File [file gkag389_supplemental_file.pdf]

Supplementary Information

HM-DyadCap – Capture and Mapping of 5-Hydroxymethyl-cytosine/5-Methylcytosine CpG Dyads in Mammalian DNA

Lena Engelhard[a§], Damian Schiller[a§], Marlon Zambrano[a,b§], Kotryna Keliuotyte[a], Benjamin Buchmuller[a], Shashank Tiwari[b], Jochen Imig[b], Angela Simeone[c], Christian Schröter[b], Sidney Becker[a,b*], and Daniel Summerer[a*]

[a]Faculty of Chemistry and Chemical Biology, TU Dortmund University, Otto-Hahn-Str. 4a, 44227 Dortmund (Germany). *Email: daniel.summerer@tu-dortmund.de and sidney.becker@mpi-dortmund.de.

[b]Max-Planck-Institute for Molecular Physiology, Otto-Hahn-Str. 11, 44227 Dortmund (Germany).

[c]Genomic Services, Qiagen, Manchester (United Kingdom).

[§]these authors contributed equally to this work

This PDF file includes:

SI Table 1-2

SI Fig. 1-17

Table S1. Oligonucleotides used in this study.

Name	Description	Sequence 5' -> 3'
o4123	qPCR, 5'-Phos	TCAGCCTTTCATTGATTGCG
o4124	qPCR	CTTCTCCTTTACTAGTGAATTC
o4368	qPCR	CTCTTCTGCCTGCTGACCTTTG
o4371	qPCR	CTTTCATTGATTGCGGATTCCAGAATTCACTAGTAAAGGAGAAGTTGGCTACAGCAA
o4372	qPCR	ACCACCCTGTTGCTGTAGCCAA
o4373	qPCR	CTTTCATTGATTGCGCACGATAGAATTCACTAGTAAAGGAGAAGGAACCGCTCATTG
o4374	qPCR	CACCATTGGCAATGAGCGGTTC
o4392	qPCR	CTTTCATTGATTGCGTCACAGAGAATTCACTAGTAAAGGAGAAGTGTAGCCCTCTGT
o4393	qPCR	CTTGAGCACACAGAGGGCTACA
o4538	NGS adapter	ACACTCTTTCCCTACACGACGCTCTTCCGATCTCTTCCTGGCACGAGTCACCCTTTCATTC ATTCCC
o4539	NGS adapter	CCACGAGATAAGAGGATGGCAAACAGCTATGACNNNNNNNNNNCTTCTCCTTTACTACTCA ATTC
o4627	template for o4372	CTCTTCTGCCTGCTGACCTTTGTCAGCCTTTCATTGATTGCGGATTCCAGAATTCACTAGT AAAGGAGAAGTTGGCTACAGCAA
o4628	template for o4374	CTCTTCTGCCTGCTGACCTTTGTCAGCCTTTCATTGATTGCGGATTCCAGAATTCACTAGT AAAGGAGAAGTTGGCTACAGCAA
o4629	template for o4393	CTCTTCTGCCTGCTGACCTTTGTCAGCCTTTCATTGATTGCGTCACAGAGAATTCACTAGT AAAGGAGAAGTGTAGCCCTCTGT
o4675	probe;<br>N,N,N,N=5hmC;<br>5'-FAM	TCTTCNGTTTCCTCAGCNGAAGGCTCGAGTCTTCNGTTTCCAAGCTTCAGCNGAAGGCTCT TCTGCCTGCTGACCTTTG
o4677	probe,<br>N,N,N,N=5mC;<br>5'-FAM	TCTTCNGTTTCCTCAGCNGAAGGCTCGAGTCTTCNGTTTCCAAGCTTCAGCNGAAGGCTCT TCTGCCTGCTGACCTTTG
o4727	probe;<br>N,N,N,N=5mC;<br>5'-Phos	CAAAGGTCAGCAGGCAGAAGAGCCTTNGGCTGAAGCTTGGAAANGGAAGACTCGAGCCTTN GGCTGAGGAAANGGAAGA
o4728	probe	TCTTCCGTTTCCTCAGCCGAAGGCTCGAGTCTTCCGTTTCCAAGCTTCAGCCGAAGGCTCT TCTGCCTGCTGACCTTTG
o4729	probe	CAAAGGTCAGCAGGCAGAAGAGCCTTCGGCTGAAGCTTGGAAACGGAAGACTCGAGCCTTC GGCTGAGGAAACGGAAGA
o5241	8NX probe template	TCACCCTTTCATTCATTCCCTCCACACAACAACCATTCTTNNNNCGNNNNAATGTGAGGAG GGTGTTATAGAATTGAGTAGTAAAGGAGAAG
o5242	PCR primer,<br>N=biotin TEG	NTCACCCTTTCATTCATTCCCTCCACACAACAACCATTC
o5243	PCR primer	CTTCTCCTTTACTACTCAATTCTATAACACCCTCCTCAC
o2968	EMSA probe<br>competitor_A	AAAAAAAAAAAAAAAAAAAAAAAA
o2969	EMSA probe<br>competitor_T	TTTTTTTTTTTTTTTTTTTTTTTT
o3112	EMSA probe<br>X=hmC	TTTTTTTTTTTXGTTTTTTTTTTT
o3115	EMSA probe<br>5'-FAM; X=hmC	AAAAAAAAAAAXGAAAAAAAAAAA
o2909	EMSA probe<br>X=mC	TTTTTTTTTTTXGTTTTTTTTTTT
o5990	EMSA probe<br>5'-FAM; X=mC	AAAAAAAANNNXANNNAAAAAAAA
o5991	EMSA probe	TTTTTTTTNNNTGNNNTTTTTTTT

o5992	EMSA probe<br>5'-FAM; X=mC	AAAAAAAANNNXTNNNAAAAAAAA
o5993	EMSA probe	TTTTTTTTNNNAGNNNTTTTTTTT
o5994	EMSA probe<br>5'-FAM; X=mC	AAAAAAAANNNXCNNNAAAAAAAA
o5995	EMSA probe	TTTTTTTTNNNGGNNNTTTTTTTT
o5996	EMSA probe<br>5'-FAM; X=mC	AAAAAAAANNNXGNNNAAAAAAAA
o5997	EMSA probe<br>X=mC	TTTTTTTTNNNXGNNNTTTTTTTT
o5998	EMSA probe<br>5'-FAM	AAAAAAAANNNCTNNNAAAAAAAA
o5999	EMSA probe<br>5'-FAM	AAAAAAAANNNCANNNAAAAAAAA
o6000	EMSA probe<br>5'-FAM	AAAAAAAANNNCCNNNAAAAAAAA
o6001	EMSA probe<br>5'-FAM	AAAAAAAANNNCGNNNAAAAAAAA
o6002	EMSA probe	TTTTTTTTNNNCGNNNTTTTTTTT
o6003	EMSA probe<br>5'-FAM	AAAAAAAANNNAANNNAAAAAAAA
o6004	EMSA probe	TTTTTTTTNNNTTNNNTTTTTTTT
o6005	EMSA probe<br>5'-FAM	AAAAAAAANNNATNNNAAAAAAAA
o6006	EMSA probe	TTTTTTTTNNNATNNNTTTTTTTT
o6007	EMSA probe<br>5'-FAM	AAAAAAAANNNACNNNAAAAAAAA
o6008	EMSA probe	TTTTTTTTNNNGTNNNTTTTTTTT
o6009	EMSA probe<br>5'-FAM	AAAAAAAANNNTANNNAAAAAAAA
o6010	EMSA probe	TTTTTTTTNNNTANNNTTTTTTTT
o6011	EMSA probe<br>5'-FAM	AAAAAAAANNNTCNNNAAAAAAAA
o6012	EMSA probe	TTTTTTTTNNNGANNNTTTTTTTT
o6013	EMSA probe<br>5'-FAM	AAAAAAAANNNGCNNNAAAAAAAA
o6014	EMSA probe	TTTTTTTTNNNGCNNNTTTTTTTT
o6411	EMSA probe<br>5'-FAM; X=mC	AAAAAAAANNNXGNNNAAAAAAAA
o5906	EMSA probe<br>5'-FAM; X=mC	TAGGCCAXGTGGGAGG
o5907	EMSA probe<br>X=hmC	CCTCCCAXGTGGCCTA
o5918	EMSA probe	CCTCCCACGTGGCCTA
o5926	EMSA probe<br>5'-FAM; X=mC	TAGGCCAXGTGGGAGG
o5927	EMSA probe<br>X=mC	CCTCCCAXGTGGCCTA
o4277	EMSA probe<br>5'-FAM	AAAAAAAAAAATGAAAAAAAAAAA
o4278	EMSA probe<br>X=mC	TTTTTTTTTTTXATTTTTTTTTTT

Table S2. Sequencing data of all sequenced libraries.

Sample ID (File)	Library Prep Kit	Input DNA (ng)	Insert Size (bp)	Read Leng	Total Reac	Raw Bases	% Q30 Bas	GC Conten	Mapping Rate (%	Duplication Rate (%
ghmC_TAYN_CAP_1_MKDL240002982-1A_L6_L2_1.fq.gz	NEBNext Ultra II DN	250	200	150	64282894	9642434100	93,23	43,87		
ghmC_TAYN_CAP_1_MKDL240002982-1A_L6_L2_2.fq.gz	NEBNext Ultra II DN	250	200	150	64282894	9642434100	91,86	44,14	95,04	28,82177843
ghmC_TAYN_CAP_2_MKDL240002982-1A_L6_L2_1.fq.gz	NEBNext Ultra II DN	250	200	150	64896802	9734520300	93,22	43,88		
ghmC_TAYN_CAP_2_MKDL240002982-1A_L6_L2_2.fq.gz	NEBNext Ultra II DN	250	200	150	64896802	9734520300	91,79	43,96	95,14	29,2052956
ghmC_TAYN_CAP_3_MKDL240002982-1A_L6_L2_1.fq.gz	NEBNext Ultra II DN	250	200	150	63199407	9479911050	93,65	43,86		
ghmC_TAYN_CAP_3_MKDL240002982-1A_L6_L2_2.fq.gz	NEBNext Ultra II DN	250	200	150	63199407	9479911050	91,95	43,75	95,23	31,58068788
Methyl-CAP-1_R_1.fq.gz	NEBNext Ultra II DN	250	200	150	127020593	19053088950	93,4	42,58		
Methyl-CAP-1_R_2.fq.gz	NEBNext Ultra II DN	250	200	150	127020593	19053088950	90,8	42,6	96,28	24,71133967
Methyl-CAP-2_R_1.fq.gz	NEBNext Ultra II DN	250	200	150	157923080	23688462000	93,28	42,6		
Methyl-CAP-2_R_2.fq.gz	NEBNext Ultra II DN	250	200	150	157923080	23688462000	91,82	42,58	96,3	25,75834775
Methyl-CAP-3_R_1.fq.gz	NEBNext Ultra II DN	250	200	150	138393530	20759029500	93,49	42,6		
Methyl-CAP-3_R_2.fq.gz	NEBNext Ultra II DN	250	200	150	138393530	20759029500	92,25	42,61	96,38	25,48510666
TAYN-CAP-1_R_1.fq.gz	NEBNext Ultra II DN	250	200	150	184981617	27747242550	93,4	41,32		
TAYN-CAP-1_R_2.fq.gz	NEBNext Ultra II DN	250	200	150	184981617	27747242550	92,04	41,32	96,59	33,75818779
TAYN-Cap-2_R_1.fq.gz	NEBNext Ultra II DN	250	200	150	147450685	22117602750	93,27	41,31		
TAYN-Cap-2_R_2.fq.gz	NEBNext Ultra II DN	250	200	150	147450685	22117602750	92,57	41,32	96,63	32,19847944
TAYN-Cap-3_R_1.fq.gz	NEBNext Ultra II DN	250	200	150	144005396	21600809400	93,17	41,28		
TAYN-Cap-3_R_2.fq.gz	NEBNext Ultra II DN	250	200	150	144005396	21600809400	91,94	41,3	96,56	31,78881241
TAYN-input_R_1.fq.gz	NEBNext Ultra II DN	250	200	150	154237980	23135697000	93,39	41,88		
TAYN-input_R_2.fq.gz	NEBNext Ultra II DN	250	200	150	154237980	23135697000	92,78	41,88	96,65	18,67804527
wt-input_R_1.fq.gz	NEBNext Ultra II DN	250	200	150	133293660	19994049000	93,22	41,86		
wt-input_R_2.fq.gz	NEBNext Ultra II DN	250	200	150	133293660	19994049000	92,09	41,84	96,65	17,01109214
R3_WT3_merge_2.fq.gz	NEBNext Ultra II DN	250	209	150	47383768	7107565200	86,45	43,42		
R3_WT3_merge_1.fq.gz	NEBNext Ultra II DN	250	209	150	47383768	7107565200	88,11	44,59	60,88	30,37651427
R3_WT2_merge_2.fq.gz	NEBNext Ultra II DN	250	209	150	52389202	7858380300	86,59	42,98		
R3_WT2_merge_1.fq.gz	NEBNext Ultra II DN	250	209	150	52389202	7858380300	87,7	43,63	62,12	30,73286866
R3_WT1_merge_2.fq.gz	NEBNext Ultra II DN	250	209	150	53868099	8080214850	87,56	42,99		
R3_WT1_merge_1.fq.gz	NEBNext Ultra II DN	250	209	150	53868099	8080214850	88,65	44,06	61,66	29,74790317
R3_TAYNgluc3_merge_2.fq.gz	NEBNext Ultra II DN	250	285	150	82530386	12379557900	91,6	42,17		
R3_TAYNgluc3_merge_1.fq.gz	NEBNext Ultra II DN	250	285	150	82530386	12379557900	92,71	42,13	84,66	35,38286924
R3_TAYNgluc2_merge_2.fq.gz	NEBNext Ultra II DN	250	285	150	85605070	12840760500	91,94	41,96		
R3_TAYNgluc2_merge_1.fq.gz	NEBNext Ultra II DN	250	285	150	85605070	12840760500	92,74	41,89	84,58	35,38944914
R3_TAYNgluc1_merge_2.fq.gz	NEBNext Ultra II DN	250	285	150	76613797	11492069550	92,23	41,8		
R3_TAYNgluc1_merge_1.fq.gz	NEBNext Ultra II DN	250	285	150	76613797	11492069550	93,14	41,73	84,7	37,27373069
R3_TAYN3_merge_2.fq.gz	NEBNext Ultra II DN	250	209	150	45835560	6875334000	84,93	43,11		
R3_TAYN3_merge_1.fq.gz	NEBNext Ultra II DN	250	209	150	45835560	6875334000	86,63	44,96	59,48	23,90566032
R3_TAYN2_merge_2.fq.gz	NEBNext Ultra II DN	250	209	150	51778983	7766847450	85,1	43,04		
R3_TAYN2_merge_1.fq.gz	NEBNext Ultra II DN	250	209	150	51778983	7766847450	86,5	44,47	60,59	24,26766815
R3_TAYN1_merge_2.fq.gz	NEBNext Ultra II DN	250	209	150	51323295	7698494250	86,65	42,24		
R3_TAYN1_merge_1.fq.gz	NEBNext Ultra II DN	250	209	150	51323295	7698494250	87,18	42,7	62,4	24,57100711
R3_input_unmodified_merge_2.fq.gz	NEBNext Ultra II DN	250	209	150	46833400	7025010000	87,06	42,25		
R3_input_unmodified_merge_1.fq.gz	NEBNext Ultra II DN	250	209	150	46833400	7025010000	87,33	43,18	61,42	19,14342731
R3_input_gluc_merge_2.fq.gz	NEBNext Ultra II DN	250	285	150	83586786	12538017900	91,71	42,37		
R3_input_gluc_merge_1.fq.gz	NEBNext Ultra II DN	250	285	150	83586786	12538017900	92,47	42,24	83,83	28,00168322
R2_WT3_merge_2.fq.gz	NEBNext Ultra II DN	250	223	150	48608378	7291256700	88,68	43,82		
R2_WT3_merge_1.fq.gz	NEBNext Ultra II DN	250	223	150	48608378	7291256700	91,3	44,54	76,71	24,1235632
R2_WT2_merge_2.fq.gz	NEBNext Ultra II DN	250	223	150	56747427	8512114050	87,7	43,61		
R2_WT2_merge_1.fq.gz	NEBNext Ultra II DN	250	223	150	56747427	8512114050	90,09	44,14	76,66	25,18976544
R2_WT1_merge_2.fq.gz	NEBNext Ultra II DN	250	223	150	53913633	8087044950	89,03	43,55		
R2_WT1_merge_1.fq.gz	NEBNext Ultra II DN	250	223	150	53913633	8087044950	91,27	44,23	77,39	24,58028754
R2_TAYNgluc3_merge_2.fq.gz	NEBNext Ultra II DN	250	277	150	63527993	9529198950	91,39	42,49		
R2_TAYNgluc3_merge_1.fq.gz	NEBNext Ultra II DN	250	277	150	63527993	9529198950	92,94	42,52	88,54	30,72548556
R2_TAYNgluc2_merge_2.fq.gz	NEBNext Ultra II DN	250	277	150	72174316	10826147400	90,97	42,27		
R2_TAYNgluc2_merge_1.fq.gz	NEBNext Ultra II DN	250	277	150	72174316	10826147400	92,45	42,3	88,4	34,74521282
R2_TAYNgluc1_merge_2.fq.gz	NEBNext Ultra II DN	250	277	150	71073537	10661030550	91,43	42,55		
R2_TAYNgluc1_merge_1.fq.gz	NEBNext Ultra II DN	250	277	150	71073537	10661030550	93,01	42,67	88,41	32,22490836
R2_TAYN3_merge_2.fq.gz	NEBNext Ultra II DN	250	223	150	52255680	7838352000	87,65	43,39		
R2_TAYN3_merge_1.fq.gz	NEBNext Ultra II DN	250	223	150	52255680	7838352000	90,1	44,49	74,54	22,33024299
R2_TAYN2_merge_2.fq.gz	NEBNext Ultra II DN	250	223	150	49723964	7458594600	85,92	43,33		
R2_TAYN2_merge_1.fq.gz	NEBNext Ultra II DN	250	223	150	49723964	7458594600	88,27	44,29	74,37	21,10740738
R2_TAYN1_merge_2.fq.gz	NEBNext Ultra II DN	250	223	150	56928185	8539227750	87,73	43,28		
R2_TAYN1_merge_1.fq.gz	NEBNext Ultra II DN	250	223	150	56928185	8539227750	89,9	43,85	75,92	22,97079035
R2_input_unmodified_merge_2.fq.gz	NEBNext Ultra II DN	250	223	150	42387000	6358050000	85,91	43,35		
R2_input_unmodified_merge_1.fq.gz	NEBNext Ultra II DN	250	223	150	42387000	6358050000	88,27	44,76	72,7	16,22863373
R2_input_gluc_merge_2.fq.gz	NEBNext Ultra II DN	250	277	150	66584038	9987605700	89,56	43,16		
R2_input_gluc_merge_1.fq.gz	NEBNext Ultra II DN	250	277	150	66584038	9987605700	91,28	43,42	86,34	24,82145739
R1_WT3_merge_2.fq.gz	NEBNext Ultra II DN	250	200	150	40575509	6086326350	86,62	43,35		
R1_WT3_merge_1.fq.gz	NEBNext Ultra II DN	250	200	150	40575509	6086326350	89,12	45,47	64,32	27,52046698
R1_WT2_merge_2.fq.gz	NEBNext Ultra II DN	250	200	150	53160457	7974068550	87,66	42,83		
R1_WT2_merge_1.fq.gz	NEBNext Ultra II DN	250	200	150	53160457	7974068550	89,39	43,95	66,38	33,02907436
R1_WT1_merge_2.fq.gz	NEBNext Ultra II DN	250	200	150	47929501	7189425150	88,36	42,87		
R1_WT1_merge_1.fq.gz	NEBNext Ultra II DN	250	200	150	47929501	7189425150	90,51	44,82	63,94	30,94089559
R1_TAYNgluc3_merge_2.fq.gz	NEBNext Ultra II DN	250	242	150	76116333	11417449950	91,34	41,95		
R1_TAYNgluc3_merge_1.fq.gz	NEBNext Ultra II DN	250	242	150	76116333	11417449950	92,3	42,39	83,25	35,9286951
R1_TAYNgluc2_merge_2.fq.gz	NEBNext Ultra II DN	250	242	150	63949953	9592492950	89,72	42,47		
R1_TAYNgluc2_merge_1.fq.gz	NEBNext Ultra II DN	250	242	150	63949953	9592492950	91,04	43,04	81,26	33,44220422
R1_TAYNgluc1_merge_2.fq.gz	NEBNext Ultra II DN	250	242	150	66893965	10034094750	91,04	41,75		
R1_TAYNgluc1_merge_1.fq.gz	NEBNext Ultra II DN	250	242	150	66893965	10034094750	91,99	42,08	83,19	37,06214128
R1_TAYN3_merge_2.fq.gz	NEBNext Ultra II DN	250	200	150	53201776	7980266400	87,14	42,65		
R1_TAYN3_merge_1.fq.gz	NEBNext Ultra II DN	250	200	150	53201776	7980266400	89,08	44,76	61,42	24,88855753
R1_TAYN2_merge_2.fq.gz	NEBNext Ultra II DN	250	200	150	40133886	6020082900	85,98	42,78		
R1_TAYN2_merge_1.fq.gz	NEBNext Ultra II DN	250	200	150	40133886	6020082900	87,08	44,5	63,85	21,90078638
R1_TAYN1_merge_2.fq.gz	NEBNext Ultra II DN	250	200	150	48528862	7279329300	86,52	42,49		
R1_TAYN1_merge_1.fq.gz	NEBNext Ultra II DN	250	200	150	48528862	7279329300	87,7	44,05	64,96	23,60141571
R1_input_unmodified_merge_2.fq.gz	NEBNext Ultra II DN	250	200	150	30128448	4519267200	84,26	42,92		
R1_input_unmodified_merge_1.fq.gz	NEBNext Ultra II DN	250	200	150	30128448	4519267200	85,27	45,79	61,86	14,77448046
R1_input_gluc_merge_2.fq.gz	NEBNext Ultra II DN	250	242	150	68911798	10336769700	90,15	42,6		
R1_input_gluc_merge_1.fq.gz	NEBNext Ultra II DN	250	242	150	68911798	10336769700	91,24	42,94	81,26	30,70395549
Me-DIP-3_2.fq.gz	MagMeDIP-seq Pack	1000	200	150	122579688	18386953200	91,81	42,35		
Me-DIP-3_1.fq.gz	MagMeDIP-seq Pack	1000	200	150	122579688	18386953200	92,2	42,46	94,97	37,34636655
Me-DIP-2_2.fq.gz	MagMeDIP-seq Pack	1000	200	150	96283985	14442597750	89,45	42,35		
Me-DIP-2_1.fq.gz	MagMeDIP-seq Pack	1000	200	150	96283985	14442597750	92,11	42,32	94,92	38,93437539
Me-DIP-1_2.fq.gz	MagMeDIP-seq Pack	1000	200	150	130872017	19630802550	89,9	42,41		
Me-DIP-1_1.fq.gz	MagMeDIP-seq Pack	1000	200	150	130872017	19630802550	92,31	42,51	94,8	39,47851424
Input-Me-DIP_2.fq.gz	MagMeDIP-seq Pack	1000	200	150	240112268	36016840200	92,74	42,48		
Input-Me-DIP_1.fq.gz	MagMeDIP-seq Pack	1000	200	150	240112268	36016840200	93,88	42,52	96,29	14,09625648
Input-hMe-DIP_2.fq.gz	MagMeDIP-seq Pack	1000	200	150	141600132	21240019800	92,99	42,16		
Input-hMe-DIP_1.fq.gz	MagMeDIP-seq Pack	1000	200	150	141600132	21240019800	93,26	42,25	96,18	12,67786965
hMe-DIP-3_2.fq.gz	MagMeDIP-seq Pack	1000	200	150	106998663	16049799450	87,87	44,21		
hMe-DIP-3_1.fq.gz	MagMeDIP-seq Pack	1000	200	150	106998663	16049799450	88,04	44,2	92,3	20,50171105
hMe-DIP-2_2.fq.gz	MagMeDIP-seq Pack	1000	200	150	121603198	18240479700	88,28	44,32		
hMe-DIP-2_1.fq.gz	MagMeDIP-seq Pack	1000	200	150	121603198	18240479700	87,94	44,42	92,35	21,61268104
hMe-DIP-1_2.fq.gz	MagMeDIP-seq Pack	1000	200	150	122621105	18393165750	87,3	43,64		
hMe-DIP-1_1.fq.gz	MagMeDIP-seq Pack	1000	200	150	122621105	18393165750	87,12	43,67	90,85	34,76021755

hmC/mC

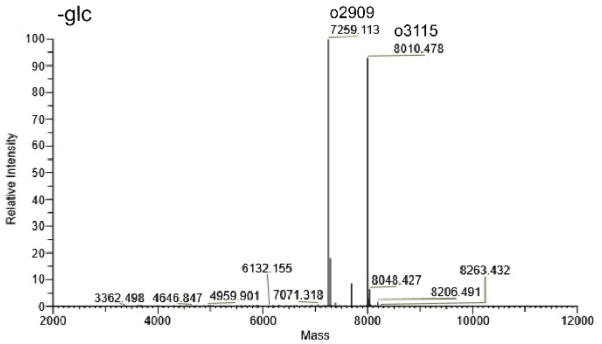

Sequence Name	Theoretical Mass, Da	Monoisotopic Mass, Da	Mass error, ppm	Relative Abundance, %
o2909	7259.172	7259.113	8.0	100
o3115	8010.540	8010.478	7.8	93.14

ghmC/mC

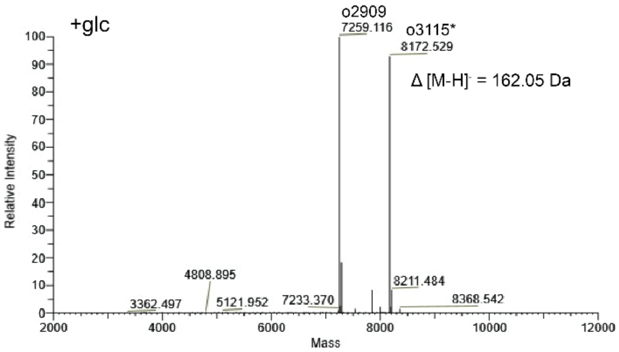

Sequence Name	Theoretical Mass, Da	Monoisotopic Mass, Da	Mass error, ppm	Relative Abundance, %
o2909	7259.172	7259.116	7.7	100
o3115	8010.540	NA	NA	NA
o3115*	8172.593	8172.529	7.8	92.93

hmC/hmC

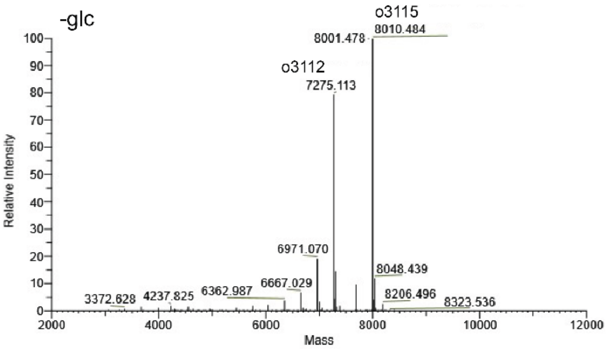

Sequence Name	Theoretical Mass, Da	Monoisotopic Mass, Da	Mass error, ppm	Relative Abundance, %
o3112	7275.167	7275.113	7.3	79.56
o3115	8010.540	8010.484	6.9	100

ghmC/ghmC

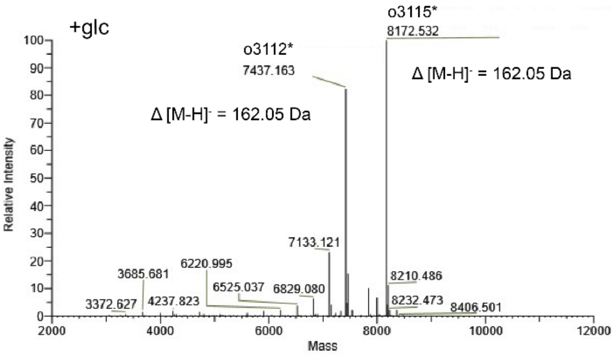

Sequence Name	Theoretical Mass, Da	Monoisotopic Mass, Da	Mass error, ppm	Relative Abundance, %
o3112*	7437.219	7437.163	7.6	82.65
o3112	7275.167	NA	NA	NA
o3115*	8172.593	8172.532	7.4	100
o3115	8010.540	NA	NA	NA

Fig. S1. T4 β-glucosyltransferase-mediated 5-hydroxymethylcytosine modification analysis using LC-MS. Deconvoluted mass spectra of hmC/mC and hmC/hmC CpG dyad containing 24-mer DNA duplexes before (left) and after (right) glucosylation.

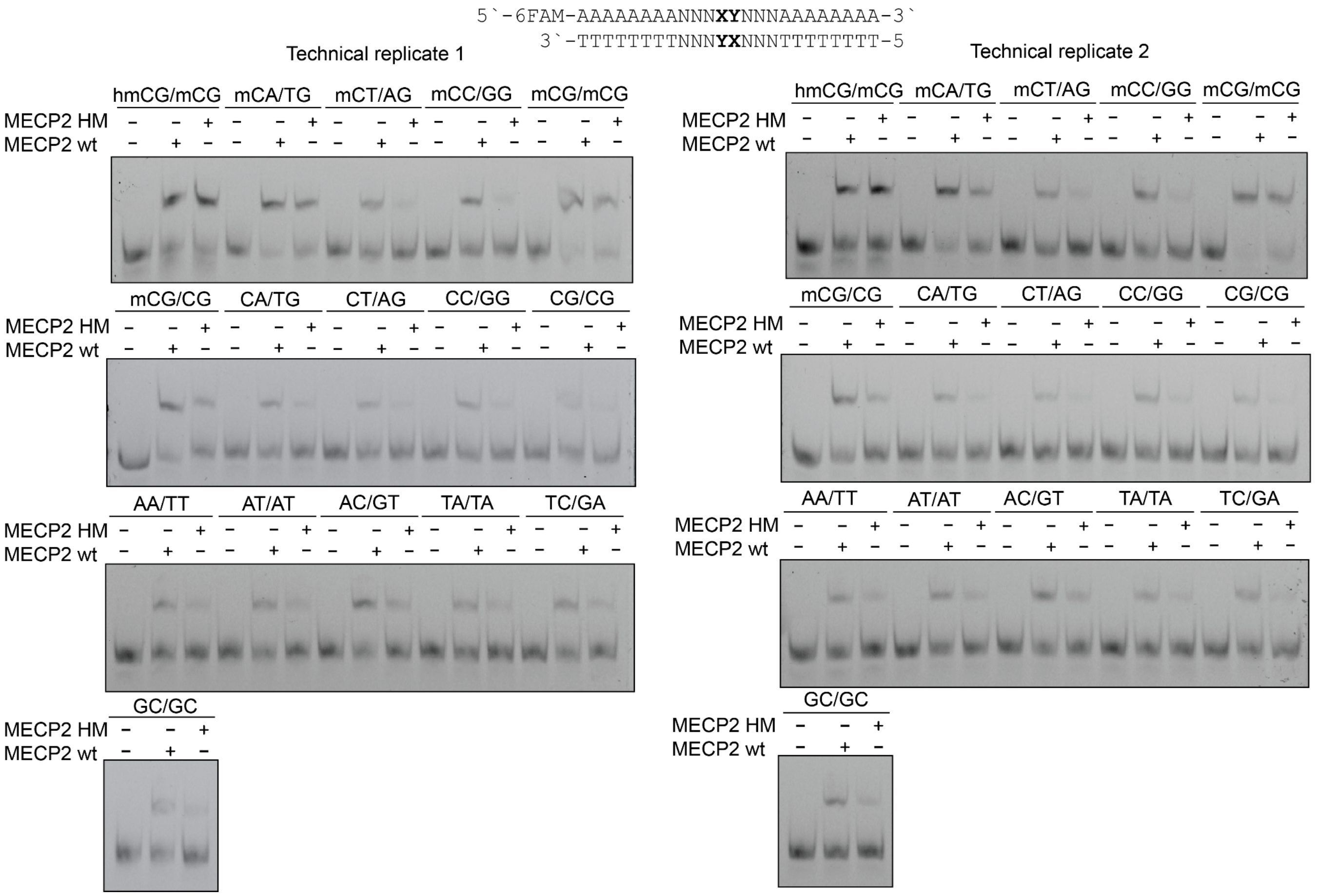

Fig. S2. Two technical replicates of electrophoretic mobility shift (EMSA) gel images showing the binding of 100 nM recombinantly expressed MECP2 HM and MECP2 wt proteins to 2 nM of 24-mer DNA duplexes containing a random sequence upstream and downstream of the indicated dinucleotides.

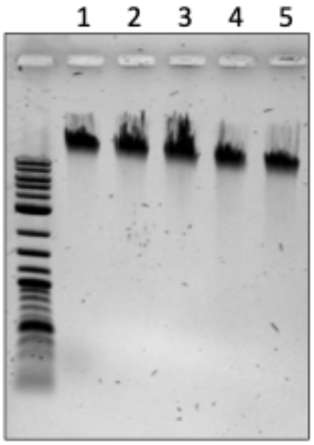

Fig. S3. Agarose gel analysis of gDNA isolated from mESC cells (E14TG2A)

Rep	input gDNA	Sheared gDNA	yield	Avg. fragment length
1	90 µg	11.2 µg	12.4 %	200 bp
2	72 µg	22.3 µg	30.9 %	223 bp
3	72 µg	13.0 µg	18.1 %	215 bp
4	72 µg	8.58 µg	11.9 %	209 bp

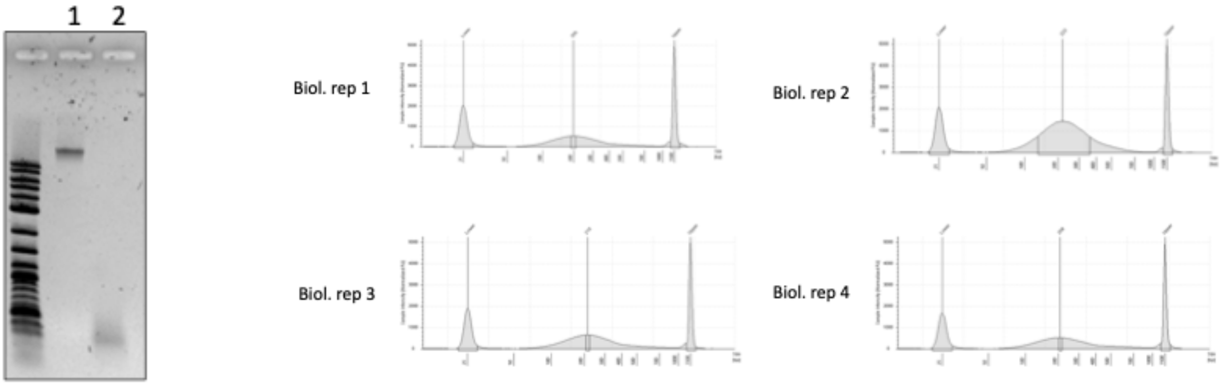

1. 10 ng/µl gDNA stock
2. Sheared DNA

Fig. S4. Agarose gel and tape station analysis of gDNA fragmentation.

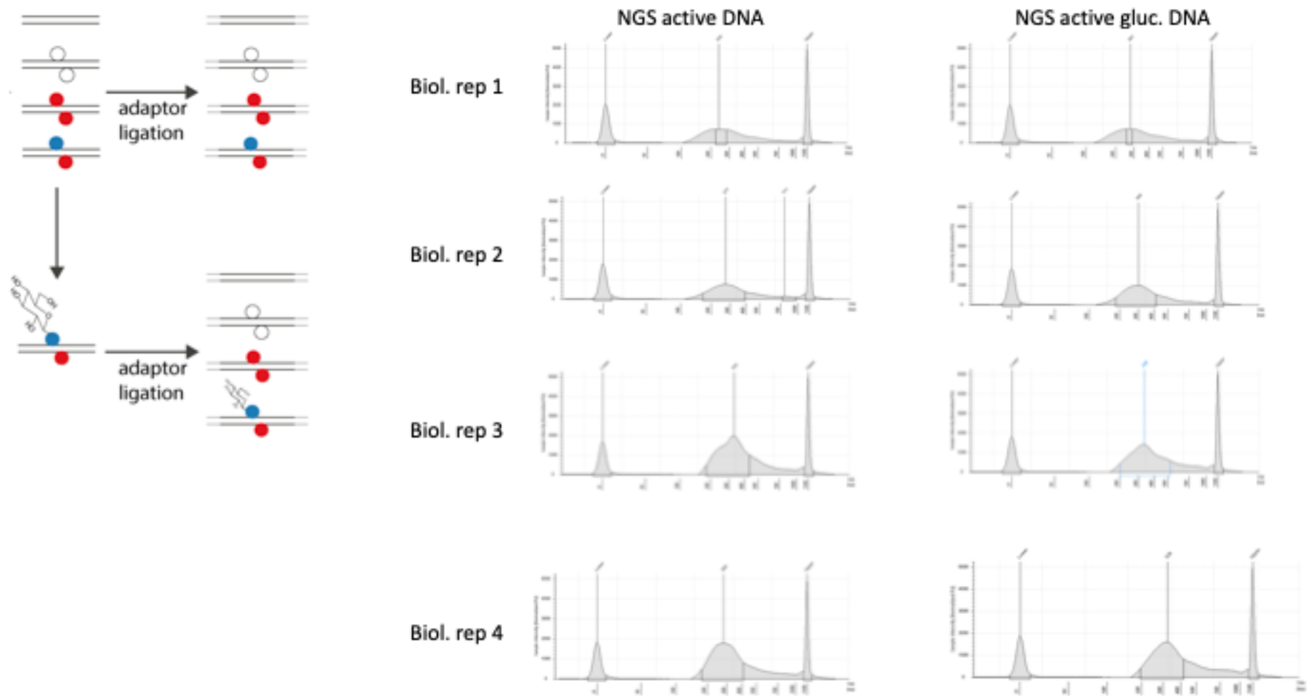

Fig. S5. Tape station analysis of adaptor ligation step to fragmented gDNA.

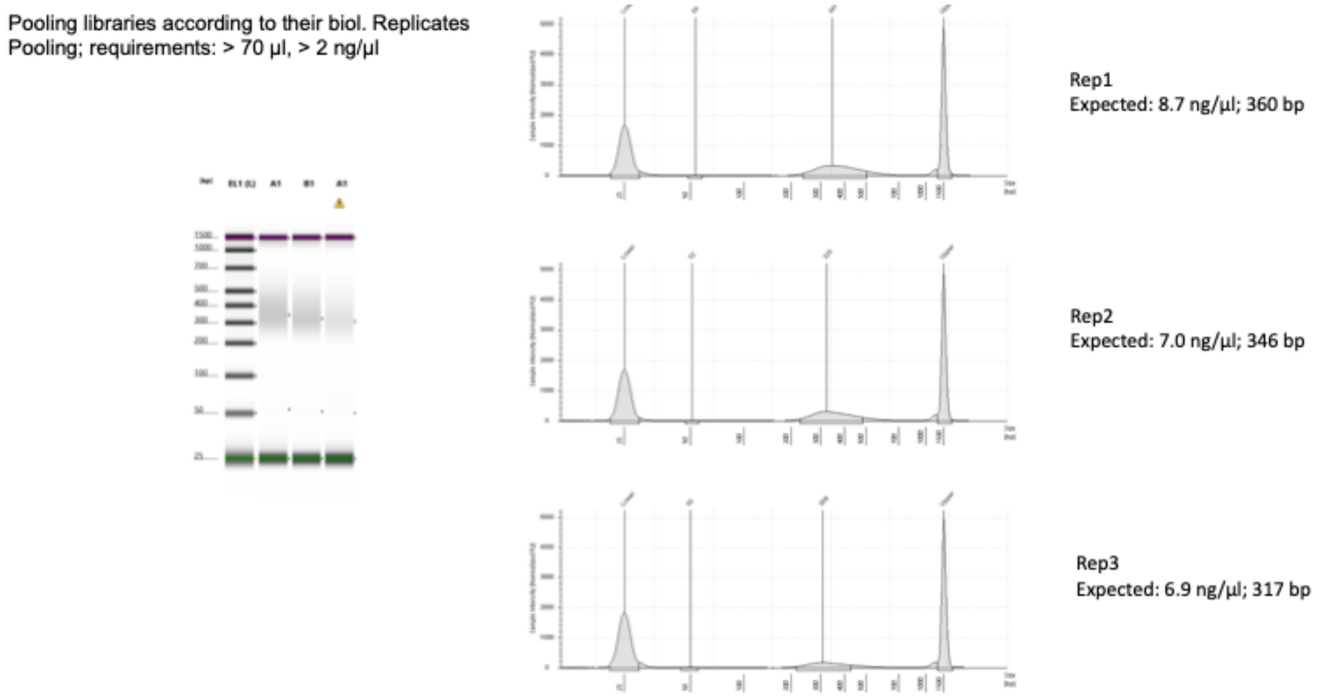

Fig. S6. Tape station analysis of final sequencing libraries of three biological replicates after pooling of barcoded technical replicates.

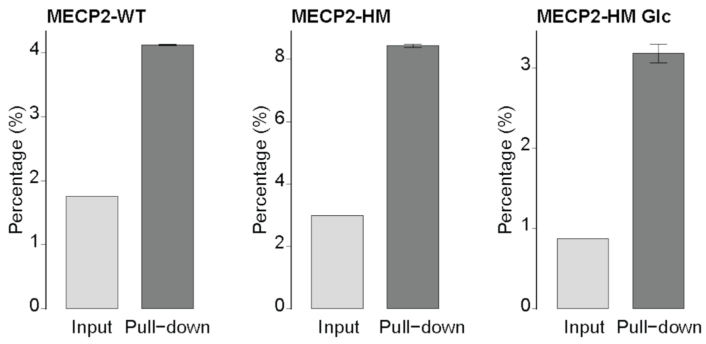

Fig. S7. Comparison of percentage of reads within MACS2-called peaks between input and captured samples across MECP2 conditions

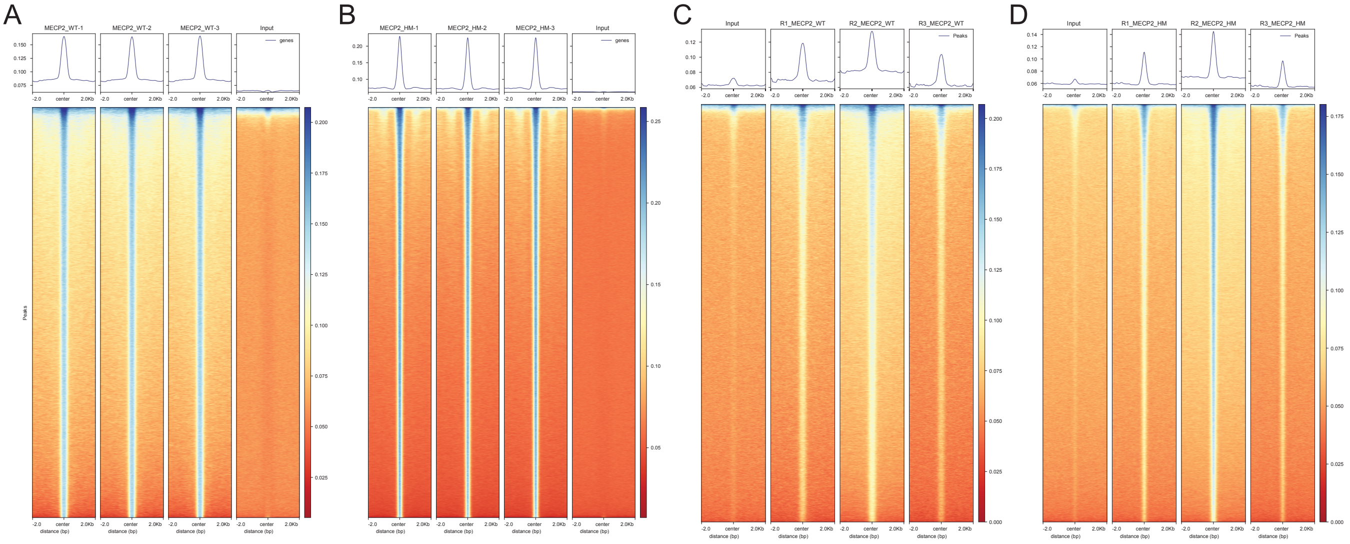

Fig S8. Enrichment profiles of MECP2-WT and -HM variants. Heatmaps and average signal profiles showing the enrichment of MECP2-WT (A, C) and MECP2-HM (B, D) around peak consensus regions (±2 kb from the enhancer center). (A, B) Technical replicates (Data set 1) and (C, D) biological replicates (Data set 2).

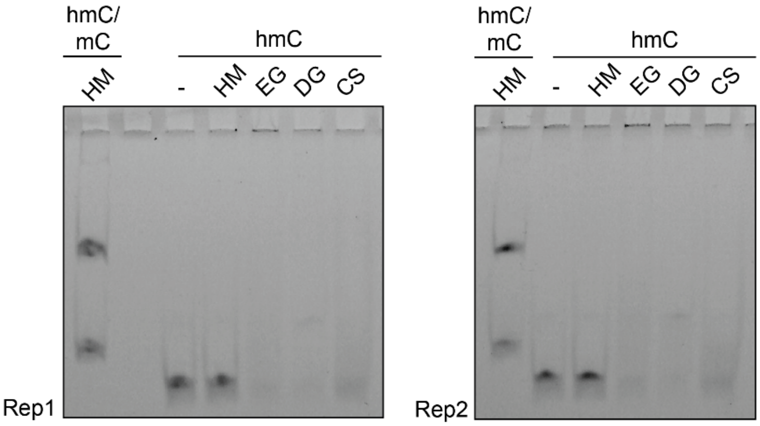

5`-6FAM-TAGGCCA**X**GTGGGAGG-3`

3`-ATCCGGTG**X**ACCCTCC-5

Fig. S9. Two technical replicates of electrophoretic mobility shift assays (EMSA) showing the binding of 100 nM recombinantly expressed MECP2 HM and three distinct monoclonal anti-hmC antibodies (EG – EpiGentek 0.1 mg/mL; DG – Diagenode 0.1 mg/mL; CS – Cell Signaling 0.02 mg/mL) to 750 pM of double-stranded (hmC/mC) or single-stranded (hmC) DNA.

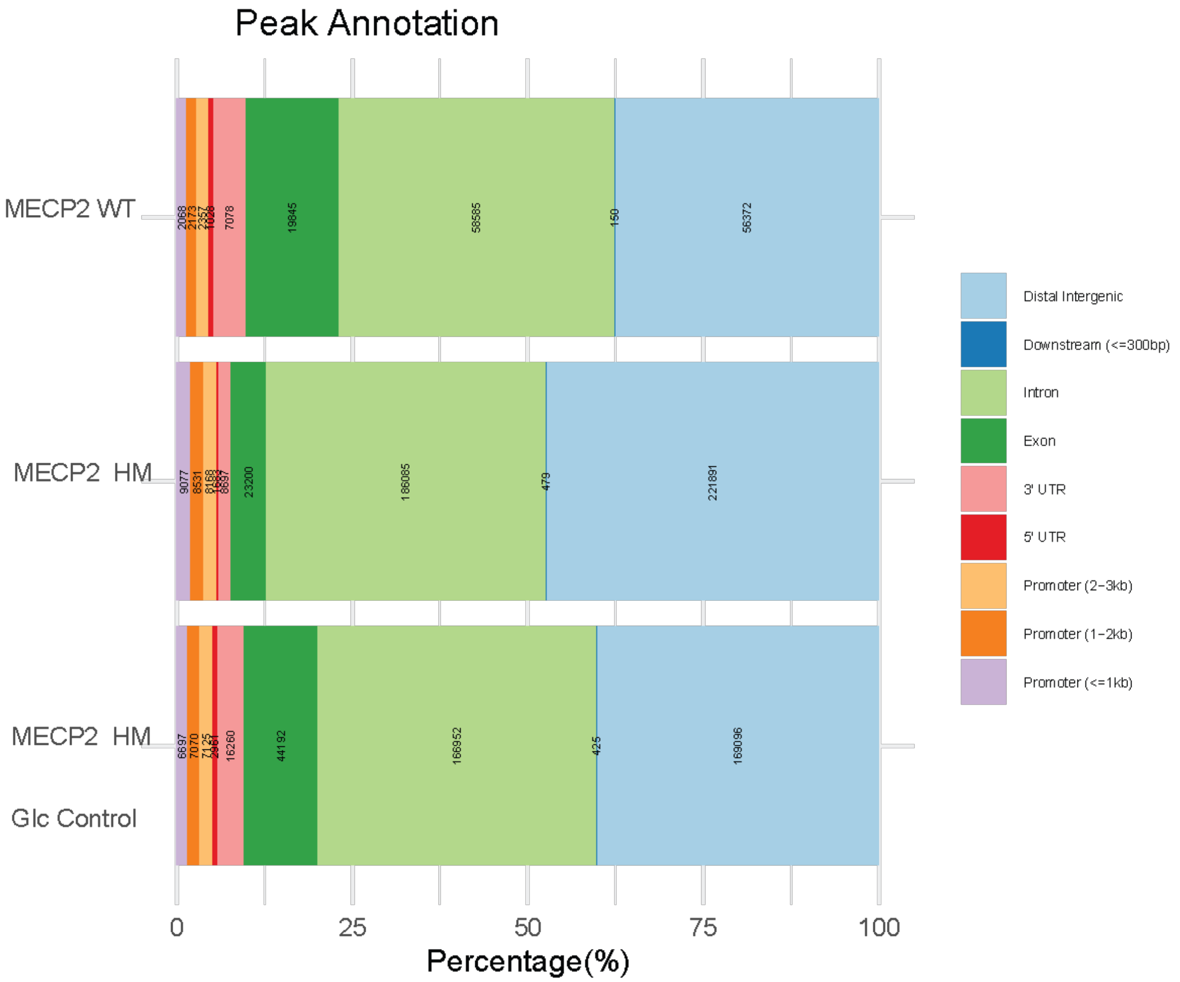

Fig. S10. Peak annotation profiles to genomic regions for MECP2 WT, MECP2 HM and MECP2 HM with glucosylated gDNA.

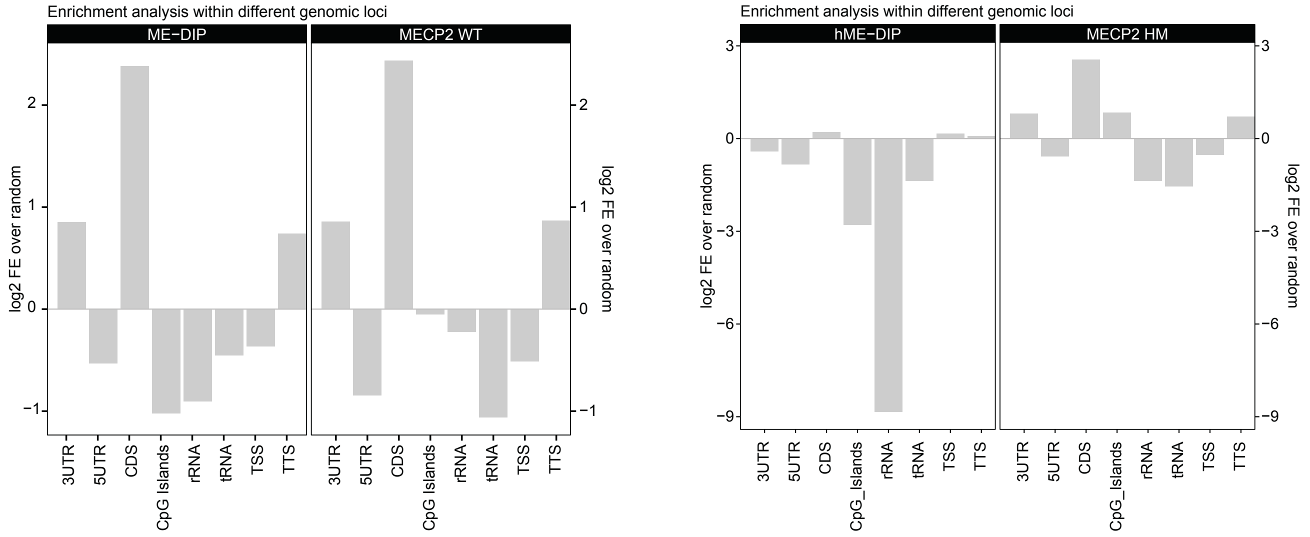

Fig. S11. Enrichment/depletion of selected genomic features for MeDIP and MECP2 wt as well as hMeDIP and MECP2_HM enrichments.

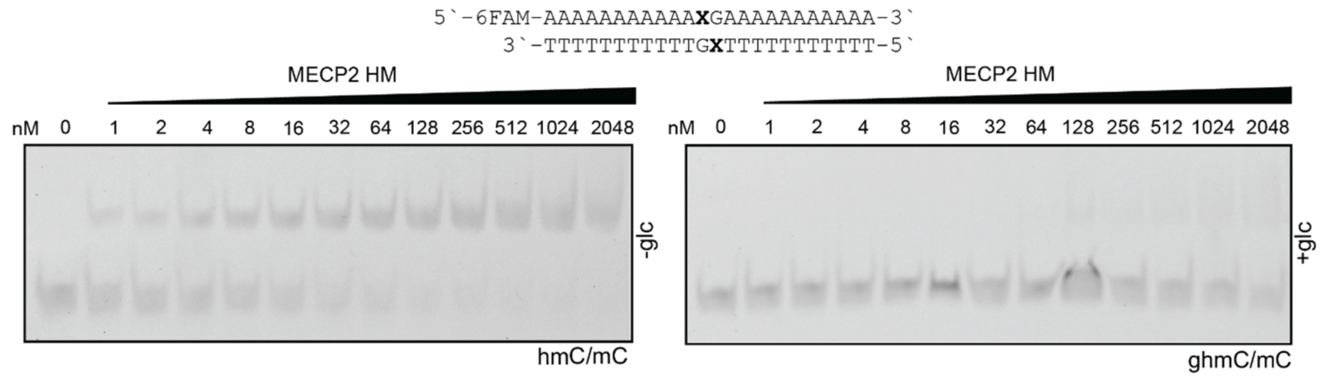

Fig. S12. Electrophoretic mobility shift assays (EMSA) showing the binding of MECP2 HM protein across dilution series with 2 nM of hmC/mC CpG dyad-containing 24-mer DNA duplexes before (left) and after (right) glucosylation.

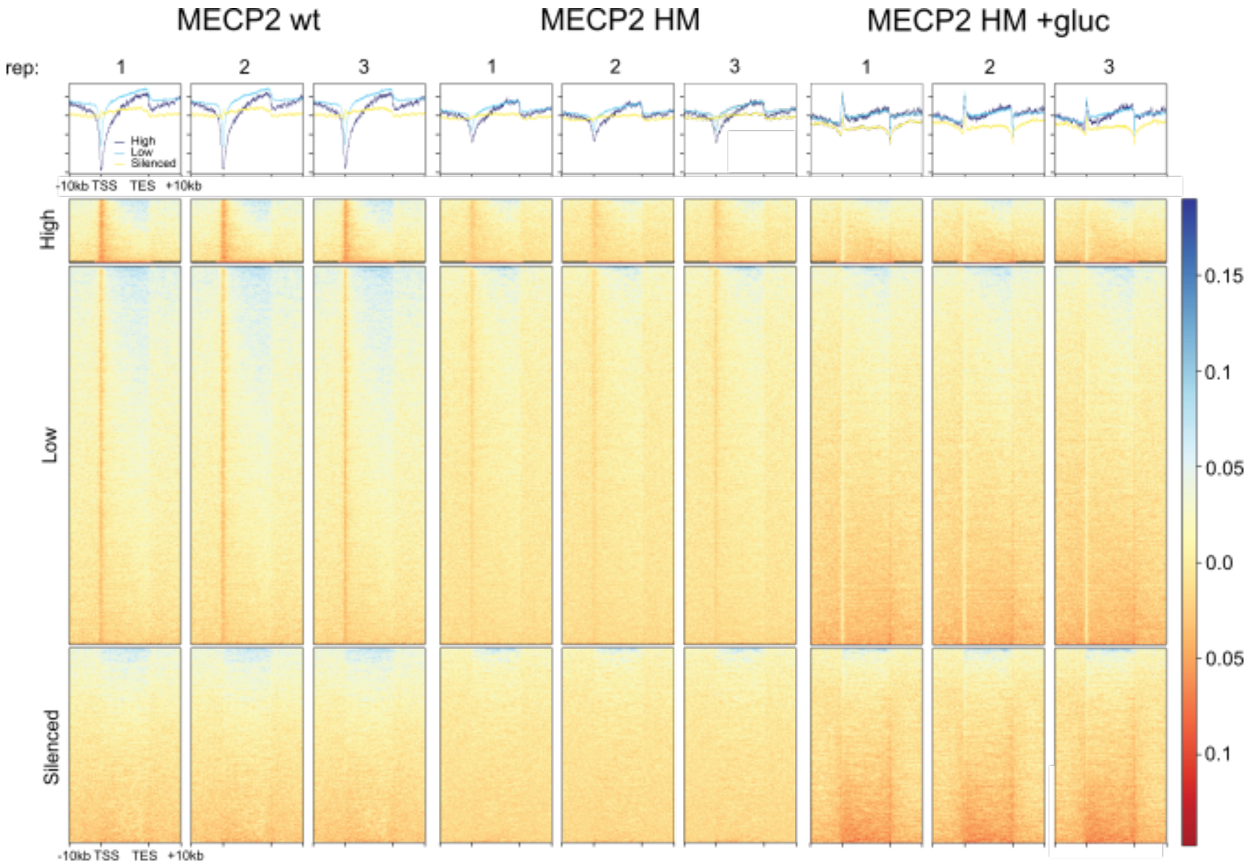

Fig. S13. Metagene read density profiles aligned to protein-coding genes, clustered according to their expression levels.

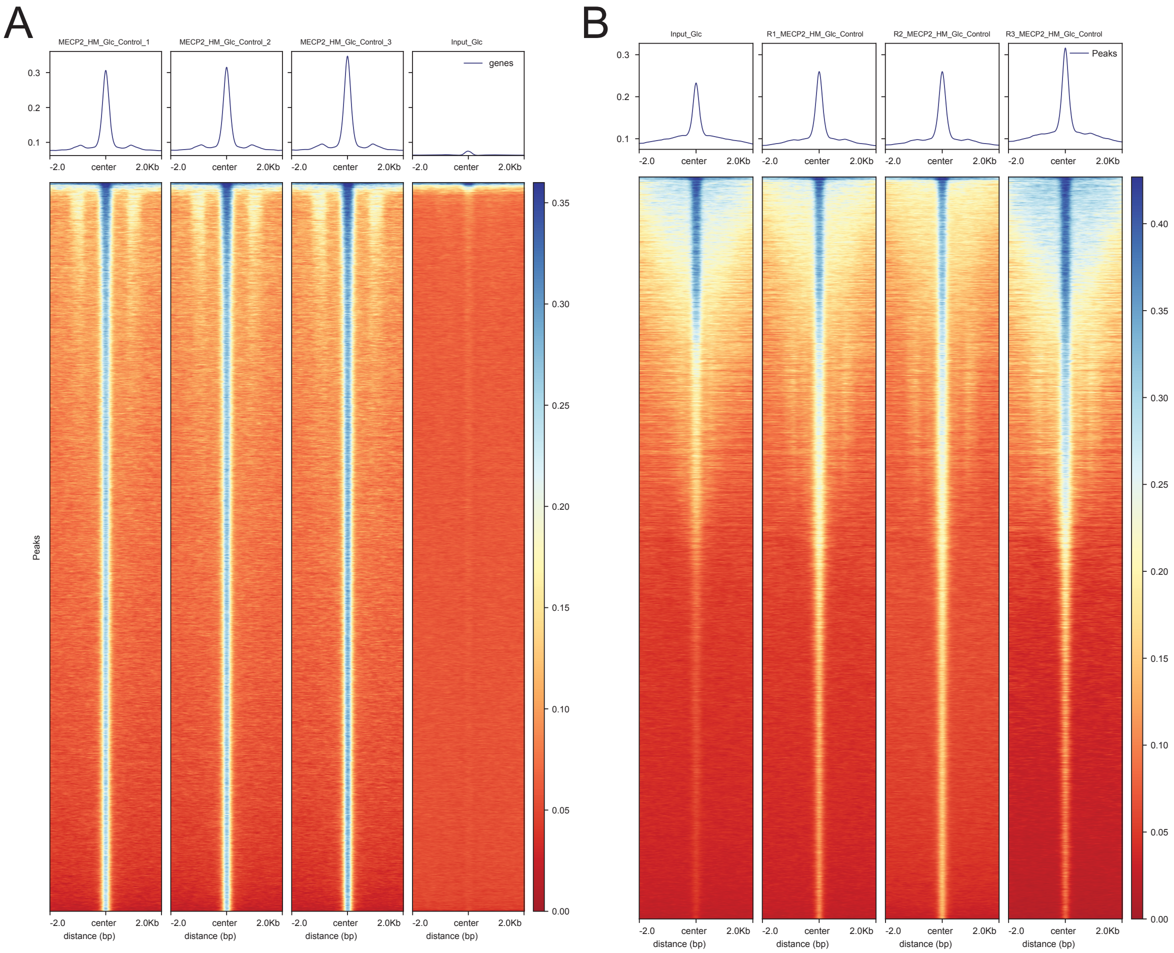

Fig S14. Enrichment profiles of MECP2-HM variant following glycosylation. Heatmaps and average signal profiles showing the enrichment of MECP2-HM-Glc around peak consensus regions (±2 kb from the enhancer center). (A) Technical replicates (Data set 1) and (B) biological replicates (Data set 2).

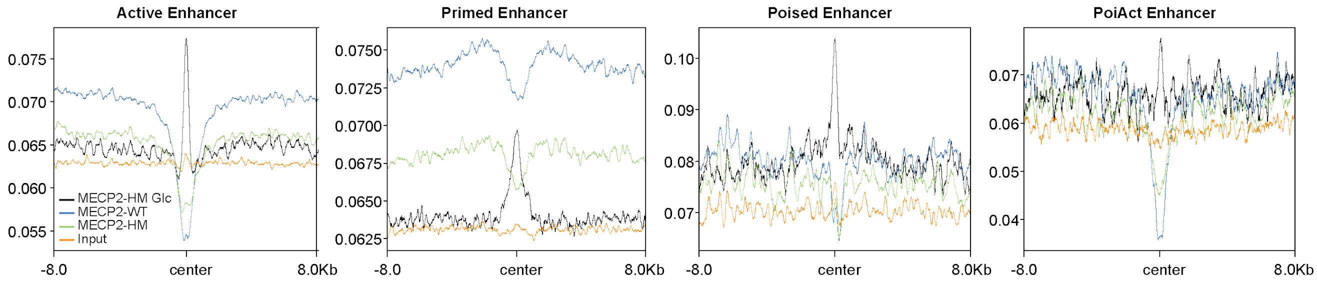

Fig. S15. MECP2 variant occupancies at four enhancer categories: active enhancers, primed enhancers, poised enhancers, and poised-to-active (PoiAct) enhancers.

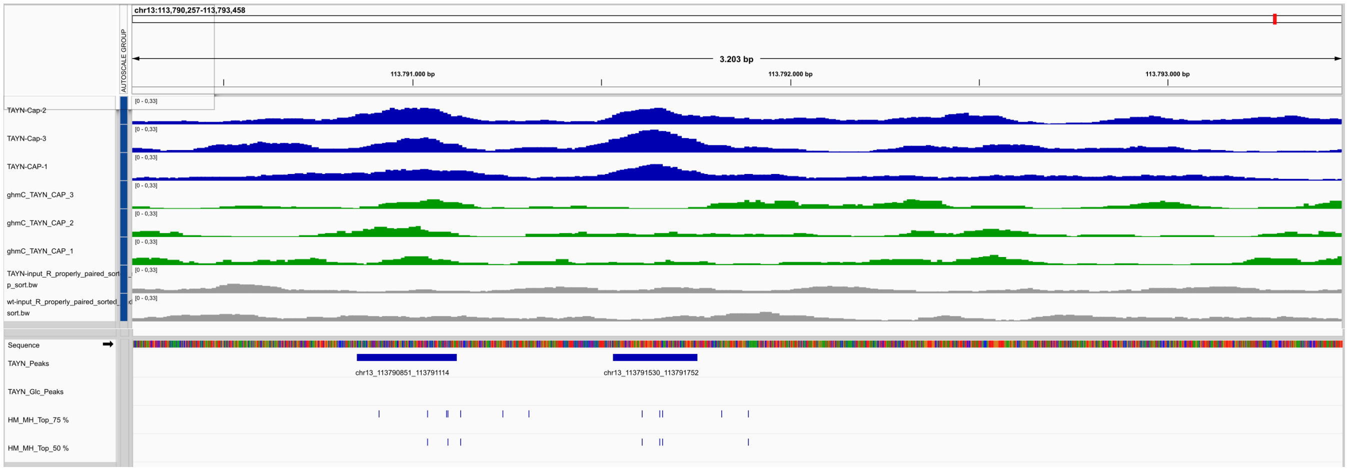

chr13:113,790,257-113,793,458
3.203 bp
113.791.000 bp
113.792.000 bp
113.793.000 bp
AUTOSCALE GROUP
TAYN-Cap-2
TAYN-Cap-3
TAYN-CAP-1
ghmC_TAYN_CAP_3
ghmC_TAYN_CAP_2
ghmC_TAYN_CAP_1
TAYN-input_R_properly_paired_sort
p_sort.bw
wt-input_R_properly_paired_sorted_
sort.bw
Sequence
TAYN_Peaks
TAYN_Glc_Peaks
HM_MH_Top_75 %
HM_MH_Top_50 %
chr13_113790851_113791114
chr13_113791530_113791752

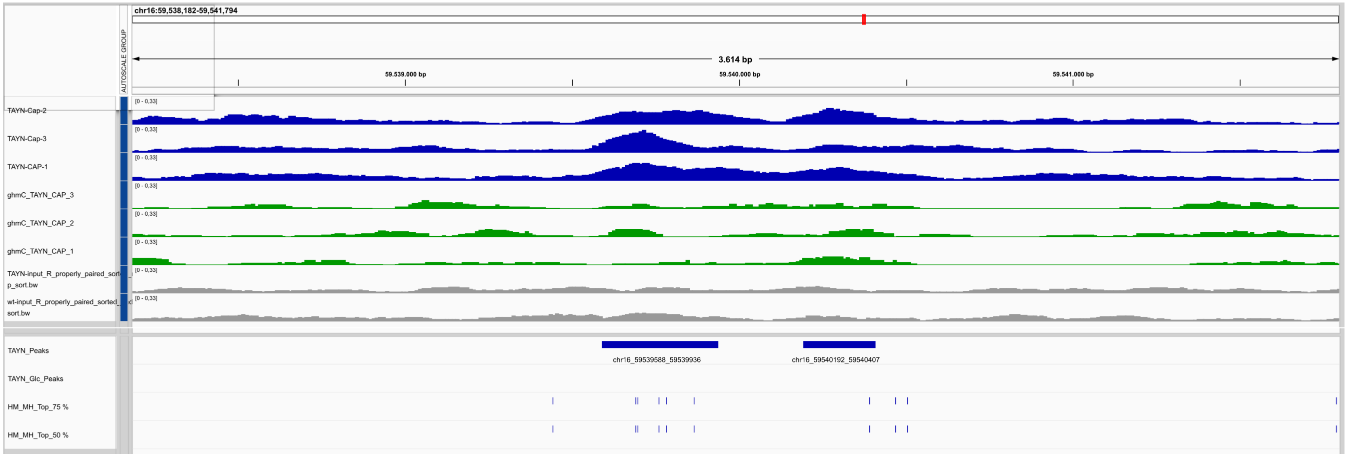

chr16:59,538,182-59,541,794
3.614 bp
59.539.000 bp
59.540.000 bp
59.541.000 bp
AUTOSCALE GROUP
TAYN-Cap-2
TAYN-Cap-3
TAYN-CAP-1
ghmC_TAYN_CAP_3
ghmC_TAYN_CAP_2
ghmC_TAYN_CAP_1
TAYN-input_R_properly_paired_sort
p_sort.bw
wt-input_R_properly_paired_sorted_
sort.bw
TAYN_Peaks
TAYN_Glc_Peaks
HM_MH_Top_75 %
HM_MH_Top_50 %
chr16_59539588_59539936
chr16_59540192_59540407

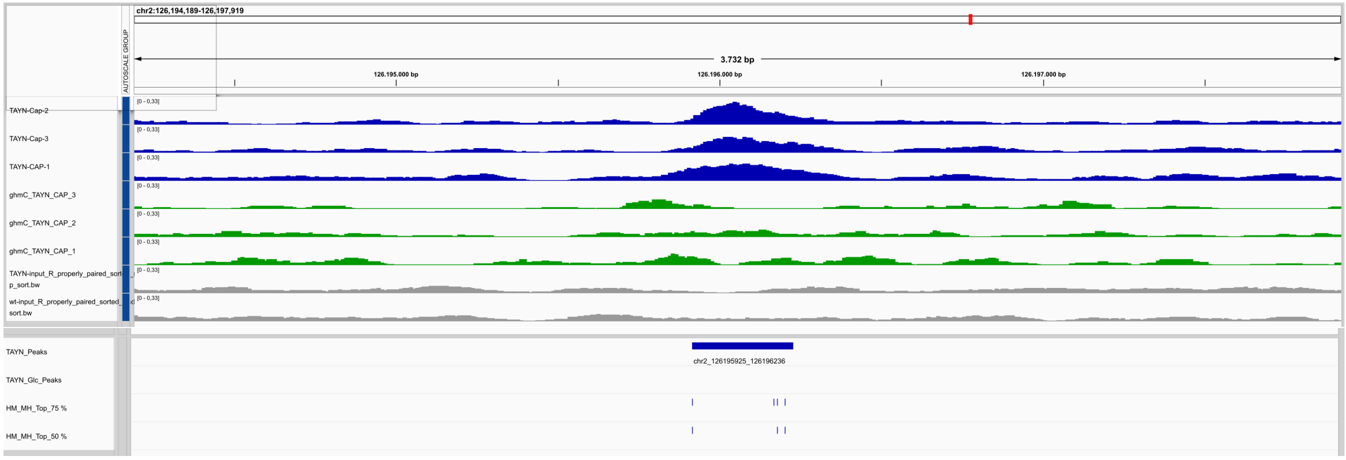

chr2:126,194,189-126,197,919
3.732 bp
126.195.000 bp
126.196.000 bp
126.197.000 bp
AUTOSCALE GROUP
TAYN-Cap-2
TAYN-Cap-3
TAYN-CAP-1
ghmC_TAYN_CAP_3
ghmC_TAYN_CAP_2
ghmC_TAYN_CAP_1
TAYN-input_R_properly_paired_sort
p_sort.bw
wt-input_R_properly_paired_sorted_
sort.bw
TAYN_Peaks
TAYN_Glc_Peaks
HM_MH_Top_75 %
HM_MH_Top_50 %
chr2_126195925_126196236

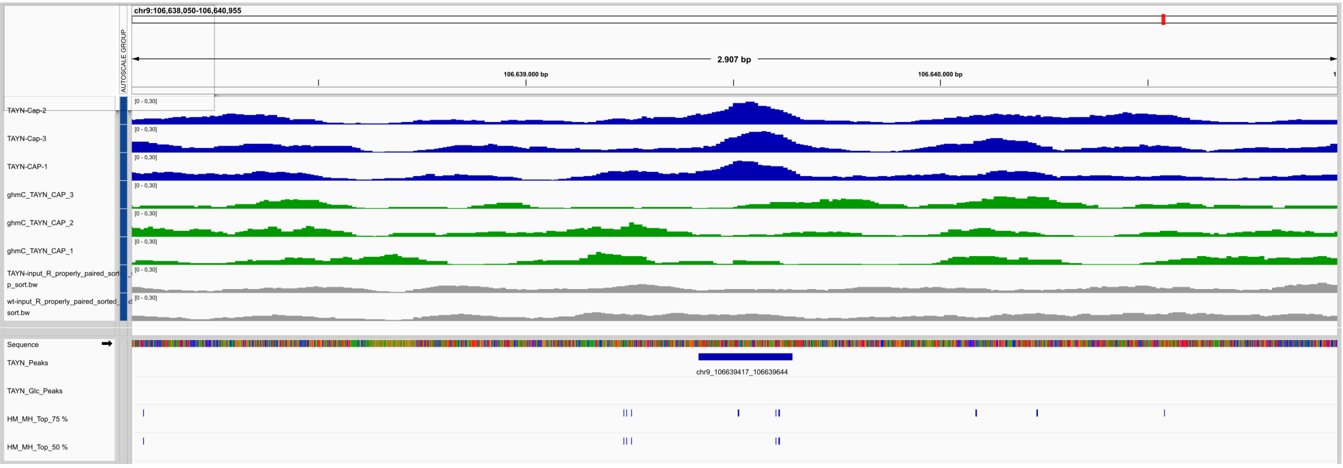
chr9:106,638,050-106,640,955
AUTOSCALE GROUP
2.907 bp
106.639.000 bp
106.640.000 bp
TAYN-Cap-2
TAYN-Cap-3
TAYN-CAP-1
ghmC_TAYN_CAP_3
ghmC_TAYN_CAP_2
ghmC_TAYN_CAP_1
TAYN-input_R_properly_paired_sort
p_sort.bw
wt-input_R_properly_paired_sorted_
sort.bw
[0 - 0,30]
Sequence
TAYN_Peaks
chr9_106639417_106639644
TAYN_Glc_Peaks
HM_MH_Top_75 %
HM_MH_Top_50 %

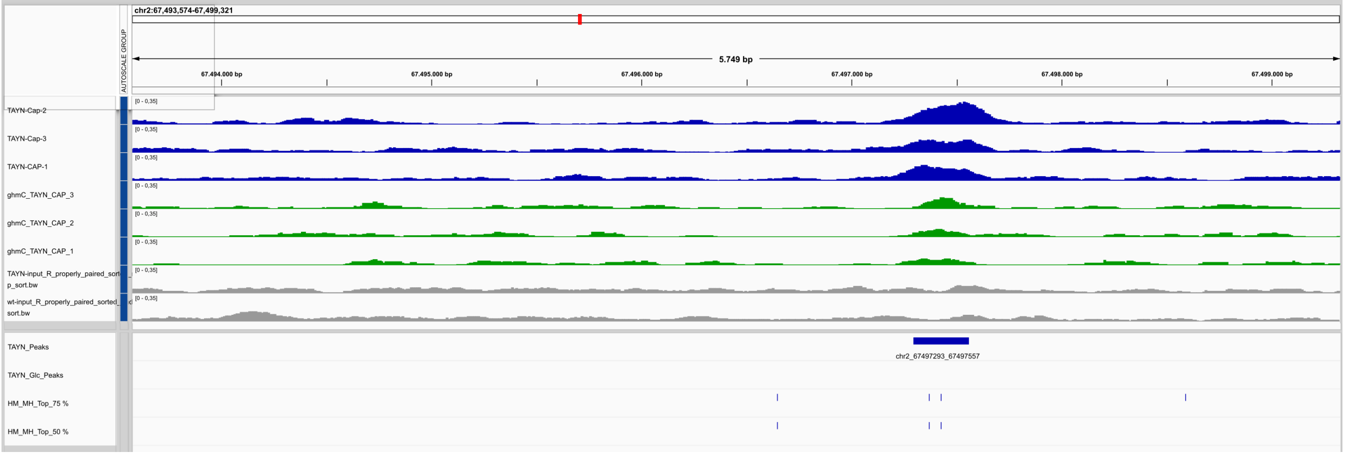
chr2:67,493,574-67,499,321
AUTOSCALE GROUP
5.749 bp
67.494.000 bp
67.495.000 bp
67.496.000 bp
67.497.000 bp
67.498.000 bp
67.499.000 bp
TAYN-Cap-2
TAYN-Cap-3
TAYN-CAP-1
ghmC_TAYN_CAP_3
ghmC_TAYN_CAP_2
ghmC_TAYN_CAP_1
TAYN-input_R_properly_paired_sort
p_sort.bw
wt-input_R_properly_paired_sorted_
sort.bw
[0 - 0,35]
TAYN_Peaks
chr2_67497293_67497557
TAYN_Glc_Peaks
HM_MH_Top_75 %
HM_MH_Top_50 %

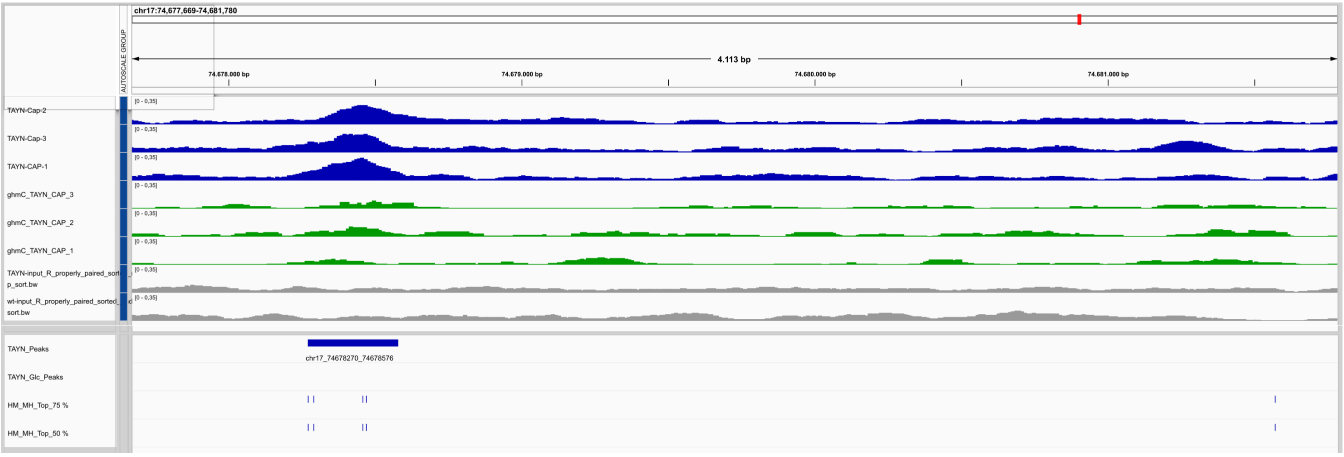
chr17:74,677,669-74,681,780
AUTOSCALE GROUP
4.113 bp
74.678.000 bp
74.679.000 bp
74.680.000 bp
74.681.000 bp
TAYN-Cap-2
TAYN-Cap-3
TAYN-CAP-1
ghmC_TAYN_CAP_3
ghmC_TAYN_CAP_2
ghmC_TAYN_CAP_1
TAYN-input_R_properly_paired_sort
p_sort.bw
wt-input_R_properly_paired_sorted_
sort.bw
[0 - 0,35]
TAYN_Peaks
chr17_74678270_74678576
TAYN_Glc_Peaks
HM_MH_Top_75 %
HM_MH_Top_50 %

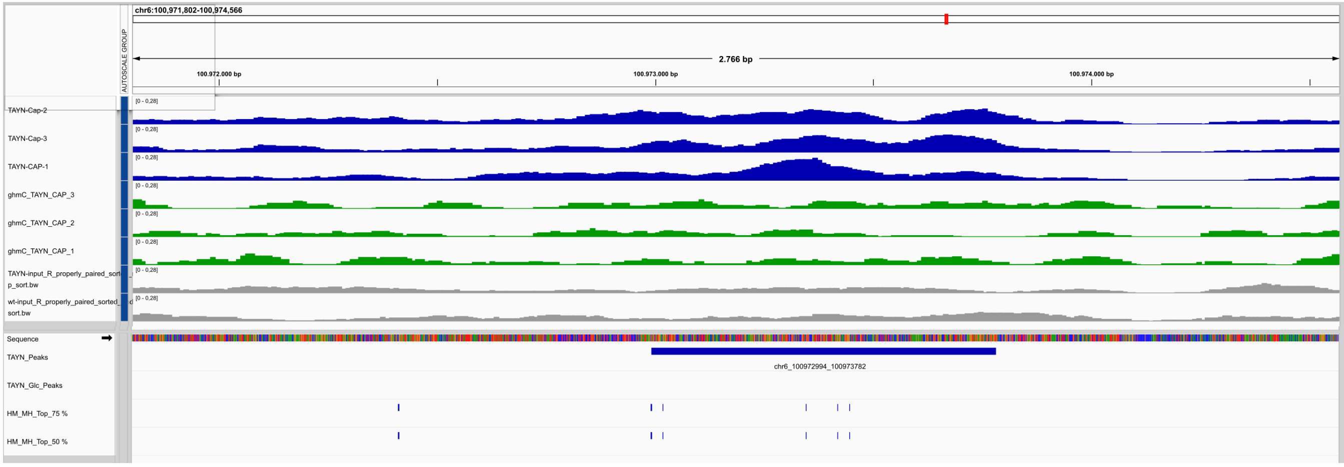
chr6:100,971,802-100,974,566
AUTOSCALE GROUP
2.766 bp
100.972.000 bp
100.973.000 bp
100.974.000 bp
TAYN-Cap-2
TAYN-Cap-3
TAYN-CAP-1
ghmC_TAYN_CAP_3
ghmC_TAYN_CAP_2
ghmC_TAYN_CAP_1
TAYN-input_R_properly_paired_sort
p_sort.bw
wt-input_R_properly_paired_sorted_
sort.bw
[0 - 0.28]
Sequence
TAYN_Peaks
TAYN_Glc_Peaks
HM_MH_Top_75 %
HM_MH_Top_50 %
chr6_100972994_100973782

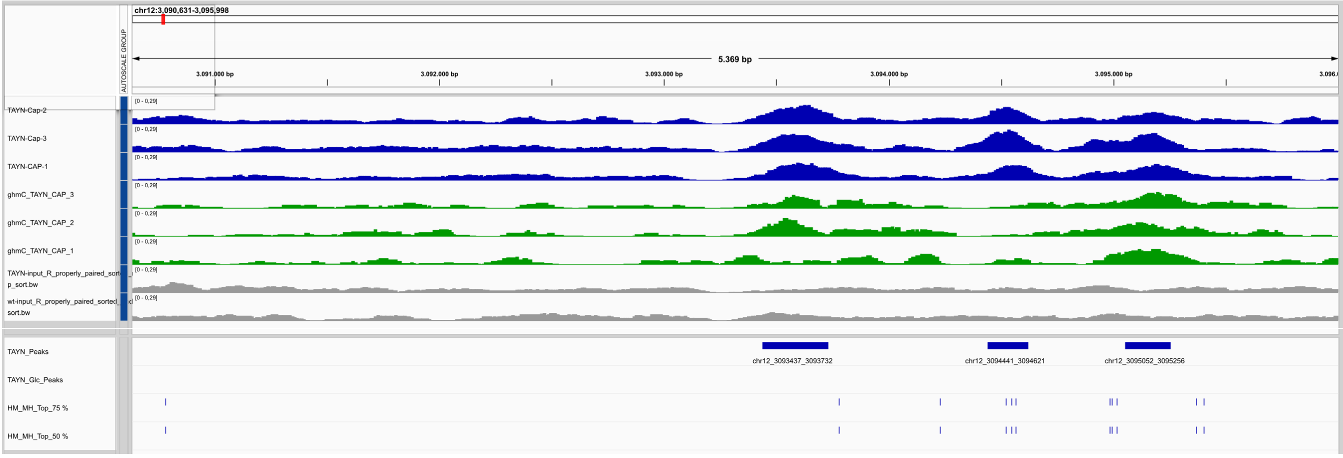
chr12:3,090,631-3,095,998
AUTOSCALE GROUP
5.369 bp
3.091.000 bp
3.092.000 bp
3.093.000 bp
3.094.000 bp
3.095.000 bp
TAYN-Cap-2
TAYN-Cap-3
TAYN-CAP-1
ghmC_TAYN_CAP_3
ghmC_TAYN_CAP_2
ghmC_TAYN_CAP_1
TAYN-input_R_properly_paired_sort
p_sort.bw
wt-input_R_properly_paired_sorted_
sort.bw
[0 - 0.29]
TAYN_Peaks
TAYN_Glc_Peaks
HM_MH_Top_75 %
HM_MH_Top_50 %
chr12_3093437_3093732
chr12_3094441_3094621
chr12_3095052_3095256

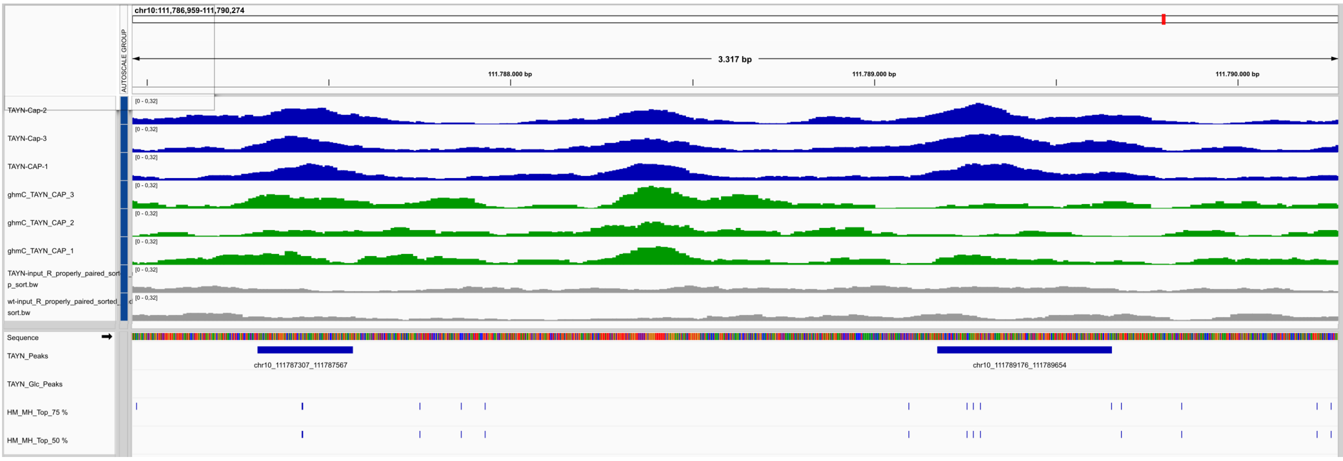
chr10:111,786,959-111,790,274
AUTOSCALE GROUP
3.317 bp
111.788.000 bp
111.789.000 bp
111.790.000 bp
TAYN-Cap-2
TAYN-Cap-3
TAYN-CAP-1
ghmC_TAYN_CAP_3
ghmC_TAYN_CAP_2
ghmC_TAYN_CAP_1
TAYN-input_R_properly_paired_sort
p_sort.bw
wt-input_R_properly_paired_sorted_
sort.bw
[0 - 0.32]
Sequence
TAYN_Peaks
TAYN_Glc_Peaks
HM_MH_Top_75 %
HM_MH_Top_50 %
chr10_111787307_111787567
chr10_111789176_111789654

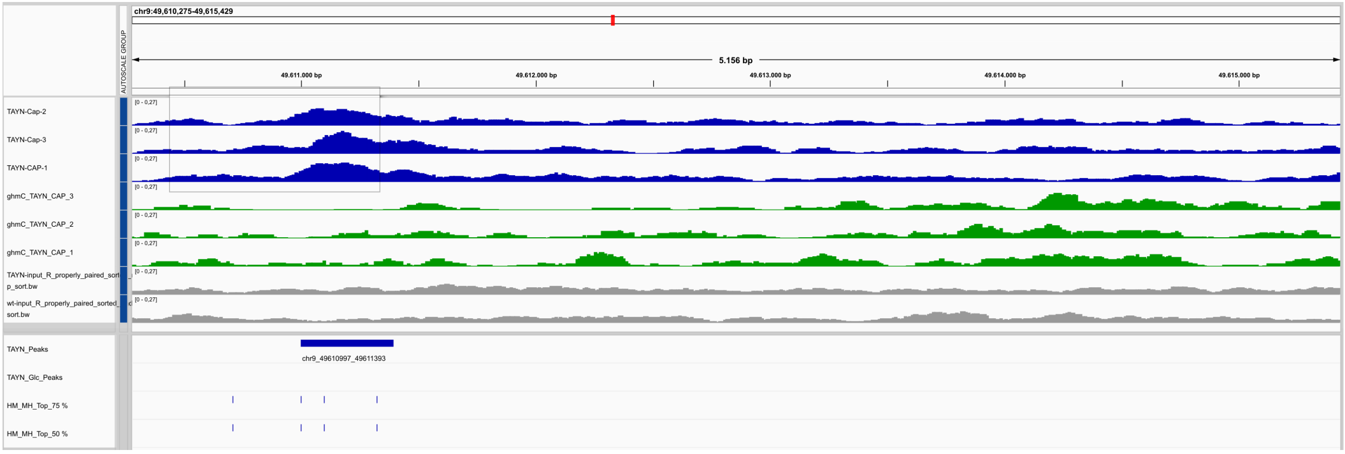

Fig. S16. Example regions of MECP2 HM enrichment signal tracks for non-glucosylated (blue) and glucosylated gDNA (green; shown are three technical replicates each. Non-enriched input DNA is shown in grey (duplicates). Regions classified as hmC/mC peaks are shown as blue bars (top: enrichment with non-glucosylated DNA; bottom: with glucosylated DNA). On the bottom, hmC/mC as well as mC/hmC sites from SCoTCH-Seq are shown as blue bars with top row showing the sites falling into the 75% most highly modified sites and bottom row falling into the 50% most highly modified sites.

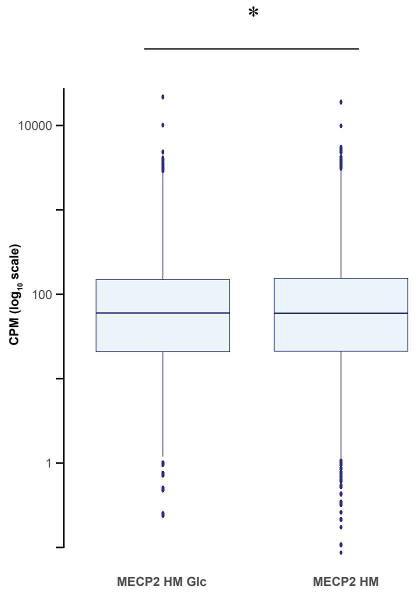

Fig. S17. Distribution of normalized read counts (CPM) across gene bodies for MECP2 HM and MECP2 HM glucosylated samples. The terminal 20% of the region downstream of the transcription start site (TSS) was excluded from the analysis. Replicates were pooled within each condition. Group differences were evaluated using the Wilcoxon rank-sum test, showing a statistically significant difference in medians (W = 2,153,144,200; p = 0.0398)
